# The haustorium: The root of biotrophic fungal pathogens

**DOI:** 10.3389/fpls.2022.963705

**Published:** 2022-08-29

**Authors:** Johannes Mapuranga, Lirong Zhang, Na Zhang, Wenxiang Yang

**Affiliations:** College of Plant Protection, Technological Innovation Center for Biological Control of Plant Diseases and Insect Pests of Hebei Province, Hebei Agricultural University, Baoding, China

**Keywords:** haustorium, pathogen, host, nutrient uptake, effectors

## Abstract

Biotrophic plant pathogenic fungi are among the dreadful pathogens that continuously threaten the production of economically important crops. The interaction of biotrophic fungal pathogens with their hosts necessitates the development of unique infection mechanisms and involvement of various virulence-associated components. Biotrophic plant pathogenic fungi have an exceptional lifestyle that supports nutrient acquisition from cells of a living host and are fully dependent on the host for successful completion of their life cycle. The haustorium, a specialized infection structure, is the key organ for biotrophic fungal pathogens. The haustorium is not only essential in the uptake of nutrients without killing the host, but also in the secretion and delivery of effectors into the host cells to manipulate host immune system and defense responses and reprogram the metabolic flow of the host. Although there is a number of unanswered questions in this area yet, results from various studies indicate that the haustorium is the root of biotrophic fungal pathogens. This review provides an overview of current knowledge of the haustorium, its structure, composition, and functions, which includes the most recent haustorial transcriptome studies.

## Introduction

Naturally, the growth and development of plants is constantly threatened by various organisms including fungi, oomycetes, viruses, and bacteria. Biotrophic fungal pathogens are undisputedly among the most intriguing category of these organisms (Voegele et al., 2009; Dean et al., 2012). Various fungi use a variety of methods to infect and invade plants. Rusts and powdery mildews are among the 10 pathogens considered most significant internationally to plant pathology (Dean et al., 2012). Powdery mildews and rust fungi consist of approximately 900 species (Braun and Cook, 2012) and over 8,000 species, respectively (Aime et al., 2014; Lorrain et al., 2019). Biotrophic pathogenic fungi cause rust diseases, which significantly affect the production of economically important crops. Stem rust, leaf rust, and yellow rust, all caused by the rust pathogens *Puccinia graminis* f. sp. *tritici* (*Pgt*), *Puccinia triticina* (*Pt*), and *Puccinia striiformis* f. sp. *tritici* (*Pst*), respectively, continue to endanger worldwide wheat production on a year-round basis (McIntosh et al., 1995; Dean et al., 2012; Hafeez et al., 2021; Kokhmetova et al., 2021). *Melampsora lini*, *Phakopsora pachyrhizi*, *Hemileia vastatrix*, and *Melampsora larici-populina*, cause flax rust, Asian soybean rust, coffee rust, and defoliating poplar rust disease, respectively (Lawrence et al., 2007; Kelly et al., 2015). During the course of an infection, plants are equipped with the ability to detect the presence of pathogens at many levels (Jones and Dangl, 2006), and as a consequence, the host defense system is activated. Pathogen-associated molecular patterns (PAMPs) are recognized by pattern-recognition receptors (PRRs) on the cell membrane, which in turn trigger PAMP-triggered immunity (PTI). Establishing a dynamic parasitic relationship between the biotrophic fungi and the host is the foundation for the development of the pathogen in host plant. In order to infect the host plant successfully, biotrophic fungal plant pathogens suppress PTI components by secreting virulence factors known as effectors through haustoria and hyphae into the host cells thereby causing diseases (Martel et al., 2021). The plants in response developed a second layer of innate immunity known as effector-triggered immunity (ETI), in which the plant resistance proteins recognize corresponding avirulence factors and set off a powerful defensive response (Jones and Dangl, 2006).

Although biotrophic pathogens like rusts and powdery mildews have unique life cycles, they both possess a sophisticated infection structure called the haustorium. The haustoria emerge after cell wall penetration and are surrounded by an invaginated plant plasma membrane (Mendgen and Deising, 1993). Fungal haustorium is widely accepted as a key player in the establishment of successful pathogenesis through nutrient acquisition from the host and delivery of effector proteins into the host cells for the manipulation of host defense response and other functions (Jones and Dangl, 2006; LoPresti et al., 2015; Jaswal et al., 2020). The expression of fungal pathogenicity is caused by differentiation-dependent gene activation, which then leads to the development of specialized hyphae that are armed with the mechanisms (enzymes, cell wall modifications) necessary to infect a host plant and cause diseases. Although the fungal haustorium was first described many years ago, many unexplained concerns persist. The major unanswered questions about haustorial biology include its establishment and composition, how it evades host recognition, how it acquires nutrients from the host cells, and how secreted effectors suppress host defense responses. Analysis of haustorial functions at molecular level is now feasible through the identification and characterization of haustorial genes and proteins. Studying haustoria function will improve our knowledge on biotrophic fungus pathogenesis. Transcriptome sequencing of haustoria, germinated spores, and urediospores may help understand the metabolic roles of infection structures and prioritize possible effector genes for subsequent functional research of this obligate biotrophic fungus. This review focuses on the establishment and development of the haustorium, its composition, gene expression, mode of nutrient acquisition, and the haustorial effectors secreted into the host cell.

## Biotrophic fungal haustorium formation and development

**Figure 1:**
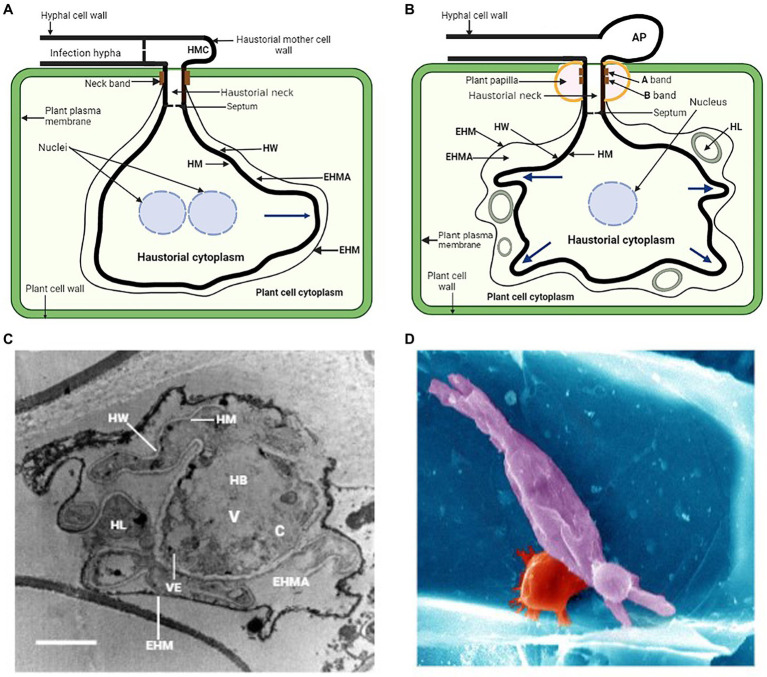
**(A)** General illustration of a rust fungus (dikaryon) haustorium and its association with the mesophyll cell of the host. HMC, haustorial mother cell; EHMA, extrahaustorial matrix; EHM, extrahaustorial membrane; HM, haustorial plasma membrane; HW, haustorial cell wall. **(B)** Schematic representation of a powdery mildew haustorial complex and its association with the epidermal cell of the host. AP, appressorium; HL, haustorial lobe. Blue arrows indicate secondary growth in these haustoria and direction of growth. **(C)** Transmission electron microscopy of a cross section of *P. xanthii* haustorial complex. The haustorial body (HB) is occupied by a large vacuole (V), a small cytoplasm (C) and many vesicles (VE). Bar, 2 μm. Picture was taken from Martínez-Cruz et al. (2014). **(D)** Scanning electron microscopy of leaf epidermal cell infected with the powdery mildew causal agent, *Blumeria* f. sp. *graminis* (false colored: barley cell wall, blue; papillae, red; fungal haustorium, purple). Picture was taken from Chowdhury et al. (2016).

The haustorium of biotrophic fungal plant pathogens adapt to the host cell’s architecture due to its morphological features. The broad morphological spectrum of haustorium is best exemplified by rust fungi which possess monokaryotic and dikaryotic stages, all of which during their infection process produce haustoria of different morphologies (Mendgen et al., 2000). Dikaryotic haustoria arise from external haustorial mother cells and consist of a slender tubular neck that penetrates into host cell and a haustorial body that forms distally to the neck (Harder and Chong, 1984; Heath and Skalamera, 1997). The haustorial mother cells thus functionally resemble the appressoria. The dikaryotic nature of fungal rust implies that they harbor significant genetic variation that is shared between the two haplotypes (Figueroa et al., 2020). Monokaryotic haustoria are terminal intercellular hyphae without morphological differentiation and have a septum near the penetration site (Gold and Mendgen, 1984, 1991; Harder and Chong, 1984). Prior to the formation of haustorium, rust fungi dikaryotic urediospores germinate on host plant epidermis and form germ tubes which sense the host cuticle topography and develop appressoria above the stomata to penetrate the intercellular spaces of the mesophyll, potentially bypassing epidermal defense responses (Mapuranga et al., 2022). Penetration occurs through stomata by the penetration hyphae into the substomatal spaces where the fungus differentiates into substomatal vesicles and elongates into an intercellular hypha which comes into contact with the host mesophyll cells and develops haustorial mother cells (Mendgen et al., 2000; Voegele et al., 2009). Following this, haustorial formation is initiated, neckbands are formed around the site of penetration of the mesophyll cell and an extrahaustorial matrix (EHMA), a gel-like layer enriched in carbohydrates, develops between the haustoria cell wall and the cell plasma membrane (Figure 1A; Staples, 2001). The extrahaustorial matrix is responsible for two functions; first to acquire nutrients like sugar and amino acids into fungal cells, and second to secrete effectors into the host cell to suppress immunity and manipulate the host. The haustorium is not directly located in the cytoplasm, although it is within the host cell, instead, it is surrounded by an extrahaustorial membrane (EHM), usually a differentiated extension of the host plant cell cytoplasm.

During haustorial formation, the host cell wall is breached and the expanding haustorium invaginates the host plasma membrane, probably by synthesizing a new membrane (Voegele and Mendgen, 2011). As the haustorial body develops, a zone of separation essential for maintaining the biotrophic lifestyle is formed between the plasma membranes of the pathogen and the host (Hahn and Mendgen, 1997). The haustorium also contains its own nuclear genes and normal complement organelles. During haustorial formation in dikaryotic rusts, migration of haustorial mother cell cytoplasmic contents, including the two haploid nuclei, into the haustorium occurs through the neck structure leaving the haustorial mother cell highly vacuolated and enucleated (Mendgen et al., 2000). Throughout host cell wall penetration and haustorial maturation, complex changes like central pore occlusion occur to the haustorial mother cell septum, and this prevents continuity of contents of the cytoplasm throughout the hyphae (Mendgen et al., 2000; Voegele and Mendgen, 2003, 2011). This suggests the separation of the haustorial mother cell and haustorium from the hyphae which probably facilitates the development of independent transcriptional and metabolic programs in these cells (Polonio et al., 2021).

Most powdery mildew fungal species use their appressoria to directly penetrate into cuticle and cell wall of the host plant to colonize exclusively the epidermal cells of the host plant (Mapuranga et al., 2022). Successful penetration results in the development of a penetration peg that penetrates directly into the epidermal cuticle of the host plant leading to the formation of haustoria which invaginates the host plasma membrane (Bushell and Bergquist, 1974). This is followed by the complete development of the haustorial body with some prolongations called haustorial lobes emerging from it (Mackie et al., 1991; Martínez-Cruz et al., 2014). Since the process of the haustorium formation causes the invagination of the host plasma membrane, which remains surrounding the haustorium and gives rise to the so-called EHM (Gil and Gay, 1977; Mackie et al., 1991; Martínez-Cruz et al., 2014), the haustorium cannot be considered a true intracellular structure (Polonio et al., 2021). The haustoria of powdery mildew are established physically inside the epidermal cells of the host and are ultimate unicellular structures consisting of a globular central body with projecting filamentous lobes (Figure 1B; Gil and Gay, 1977). There are two main morphologies of powdery mildew haustoria that have been described; the globular haustoria with projecting filamentous tubular lobes typical of Arabidopsis powdery mildew *Golovinomyces orontii* and *Podosphaera xanthii* a cucurbit powdery mildew (Figures 1B,C; Micali et al., 2011; Martínez-Cruz et al., 2014), and the highly branched multidigitate haustoria typical of barley powdery mildew *Blumeria graminis*. f. sp. *hordei* (Figure 1D; Lambertucci et al., 2019; Polonio et al., 2021). Powdery mildew appressorium and haustorium are separated by the haustorial neck where the EHM and the haustorial membrane (HM) are wrapped by two neckband regions termed A band and B band (Figure 1B; Stumpf and Gay, 1990; Mendgen et al., 2000; Martínez-Cruz et al., 2014). This neckband protects the extrahaustorial matrix (EHMA) against the bulk apoplast, resembling the endodermal Casparian strip (Heath, 1976). The haustorial cytoplasm and the appressorium are divided by a septum that has a hole and is the consequence of the rupture of a papilla (Mackie et al., 1991; Micali et al., 2011). This results in the formation of a space between the EHM and the HM that is referred to as the EHMA (Figures 1A,B; Gil and Gay, 1977). This space appears to be well-suited for the absorption of nutrition from the host and was regarded as a symplastic section (Mendgen et al., 2000; Micali et al., 2011). The haustorial body, haustorial lobes, HM, EHM, and EMHA make up the haustorial complex (HC; Mackie et al., 1991). This complex also includes the haustorial mesentery. Haustorial lobes form during haustorial maturity and the inner parts of haustorial complexes showed a haustorial body surrounded by irregular lobes (Gil and Gay, 1977; Micali et al., 2011; Martínez-Cruz et al., 2014). Transmission electron microscopy revealed an immature haustorium with a tiny haustorial body and no subcellular characteristics, and a bigger, highly organized mature haustorium (Martínez-Cruz et al., 2014). As in *G. orontii* and *B. graminis* f. sp. *hordei*, *P. xanthii* has lobes at both ends (Godfrey et al., 2009; Micali et al., 2011; Martínez-Cruz et al., 2014). Tiny lobes suggest young complexes, whereas wide lobes indicate mature complexes. Massive vacuoles and electron-dense vesicles dominated the haustorial core. Some lobes share the haustorial body’s cytoplasm and cell wall. The haustorial body and lobes were separated from the plant cytoplasm by an electron-dense membrane (Martínez-Cruz et al., 2014). Haustorial lobes are involved in *P. xanthii*-host cell communication because they cover nearly the entire haustorial body, increasing contact area and facilitating material exchange. Furthermore, they probably facilitate vesicle secretion and transit because of their cell wall which is thinner than the haustorial body. (Eichmann and Hückelhoven, 2008; Rodrigues et al., 2008; Martínez-Cruz et al., 2014). In yeasts and filamentous fungi, the vesicular pathway has been extensively studied (Fischer-Parton et al., 2000), and in these species, vesicular trafficking involves different-sized vesicles. It is unknown whether vesicular trafficking occurs in rusts, powdery mildews, or haustorium function. The cytoskeleton of the host plant goes through a process of polarization alteration throughout the process of haustorial development, which is linked to the host’s immune response (Henty-Ridilla et al., 2013; Polonio et al., 2021).

Changes in microtubule arrangement affect protein activity *via* direct or indirect transport (Schmidt and Panstruga, 2011). Papillae are one of the first plant structures that arise in response to a pathogen attack and they are made of cellulose and other polymers that curb pathogen proliferation (Wang et al., 2009; Eggert et al., 2014). This protective protein drives actin filament reorganization, which surrounds haustorial complexes and directs callose formation (Lipka and Panstruga, 2005; Eichmann and Hückelhoven, 2008). Actin filaments arrange themselves around the nucleus, bringing it closer to the point of entry in order to modify gene expression and facilitate a more rapid immune response (Eichmann et al., 2004). Fungal effectors affect plant cytoskeletons, whereas plant peptides target fungal cytoskeletons (Schmidt and Panstruga, 2007). Transmission electron microscopy (TEM) study demonstrated the very irregular shape of the EHM, which separates the EHMA of haustoria from the plant cell’s cytoplasm. TEM examination showed haustoria-surrounding vesicles and electron-dense plaques which had been deposited on the *P. xanthii* haustoria, and it is most likely that these plaques originated from the plant host (Martínez-Cruz et al., 2014). Due to papilla development, callose deposition was limited to fungal penetration sites in colony perimeters. This clearly showed that haustorial maturity is associated with callose deposition (Martínez-Cruz et al., 2014).

### Haustorial composition

The haustorium originated from a hypha as previously alluded, so it obviously appears to think that these two structures have the same composition. However, various haustorium distinct features have been explained (Mackie et al., 1991; Micali et al., 2011; Martínez-Cruz et al., 2014). For the vast majority of interactions with haustoria, where they are known as extrahaustorial matrices (EHMAs), they are also known as interfacial extracellular matrices (IFM), which refers to a specific subset of interactions. These matrices vary by organism. The homogeneous, amorphous appearance of these matrices reflects the uniform distribution of key components (Bracker and Littlefield, 1973). In haustoria, matrices are thickest around haustorial lobes and weakest, if existent, near the neck, where the host plasma membrane presses against the penetration peg cell wall (Bracker and Littlefield, 1973). There is a clear distinction between the fungal cell wall and the haustorial neckbands. Energy dispersive X-ray examination revealed a significant concentration of iron, phosphate, silicon, glucans, lipids, and proteins in the neckband of rust haustoria (Harder and Chong, 1991; Mendgen et al., 2000). The A band has a high concentration of 1,3-glucans, and the lipid molecules that make up this band are bonded to both chitin and 1,3-glucans. The B band has a high concentration of 1,4-glucans and the lipid molecules it contains are covalently bonded to its proteinaceous components (Stumpf and Gay, 1990; Martínez-Cruz et al., 2014). Maintaining an effective seal between the EHMA and the plant’s plasma membrane is thought to be critical for optimal nutrient transfer into haustoria (Hahn et al., 1997). The neckband creates a seal, hence EHMA is a contained entity, unlike apoplast (Bracker and Littlefield, 1973; Woods and Gay, 1983; Green et al., 1992). EHMA is a carbohydrate-rich layer between the haustorium and the EHM. A wide range of cytological approaches have been used to examine the EHMA composition surrounding the haustoria (Harder and Chong, 1991). *Erysiphe. pisi*’s EHMA expands in hypotonic solutions without rupturing the haustoria, indicating they are fluid (Gil and Gay, 1977). Small compounds like uranyl ions may get through the EHMA, but horse radish peroxidase cannot. The EHMA around *E. pisi* haustoria is gel-like, not a solution (Gay and Manners, 1987; Green et al., 1992).

The fungus may decrease plant cell wall formation and/or deposition at the invaginated plant plasma membrane (Green et al., 1995). EHMAs generated by obligate biotrophs infecting monocots include threonine-hydroxyproline-rich glycoproteins (THRGPs; Hippe-Sanwald et al., 1994), which may be plant-produced compounds that function as a fungal barrier (Hippe-Sanwald et al., 1994). A study of the EHMA around *Uromyces vignae* monokaryotic rust haustoria found plant hydroxyproline-rich glycoproteins (HRGPs), arabinogalactan proteins (AGPs), and callose (Stark-Urnau and Mendgen, 1995). Other components of the host cell wall such as cellulose and arabinogalactan proteins were also detected in the EHM using immunogold labeling. These findings established that the plant contributes to EHMA in certain obligatory biotrophic relationships but not in others (Stark-Urnau and Mendgen, 1995). Multivesicular bodies (MVBs) comprising lipid bilayers were discovered in the ultrastructure of *G. orontii* haustoria in another study (Micali et al., 2011). It was found that the delimitation membrane of each individual endosomal compartment is pushed inward, resulting in an increase in the volume of the endosomal lumens. In anatomically comparable haustorial lobes, there were more medium- and large-sized vesicles than in the body. MVBs have been proposed as the vehicles for transporting small vesicles to vacuoles or for the release of small vesicles called exosomes to the extracellular environment through fusion with the plasma membrane (Valadi et al., 2007; Rodrigues et al., 2008; Schorey and Bhatnagar, 2008; Théry, 2011). Small vesicles were found in the EHMA, at the EHM, and haustorial lobes, and prior findings suggested that these vesicles were exosomes discharged into the EHMA by MVB fusion with the haustorial membrane (Martínez-Cruz et al., 2014). Fungal effectors, proteins, microRNAs, and mRNAs can all be delivered by exosomes in powdery mildew haustoria, and this may be an effective method for the transport of these substances to the haustorium (Valadi et al., 2007).

The EHM is distinct from the non-invaginated section of the host plasma membrane by many utrastructural characteristics. In most cases, it is thicker than the plasma membrane and has a distinct color when stained. Cytological studies showed that the EHM has specific functional features (Harder, 1989). The EHM’s genesis is uncertain, but two hypotheses have been proposed. First, EHM is caused by fungal activity altering the plant plasma membrane over time. The EHMA is a nutrition and information trading hub (Heath and Skalamera, 1997). Freeze fracture experiments demonstrated that EHMs generated by dikaryotic stage rusts and powdery mildews lack intramembrane particles and exhibit corrugations and protuberances that increase with haustoria age (Littlefield and Bracker, 1972; Harder and Mendgen, 1982; Harder and Chong, 1984). Convolutions in invaginated membranes may help the fungus to acquire nutrients by increasing surface area (Manners and Gay, 1983). Powdery mildew EHMs seem thicker than peripheral host plasma membranes, probably because of additional carbohydrates. EHM lacks ATPase activity, unlike plant plasma membranes (Polonio et al., 2021). Antibody labeling of *E. pisi* haustoria demonstrated that the EHM shares certain glycoproteins with the host plasma membrane but lacks others (Green et al., 1995). Invading fungi modify the invaginated plasma membrane for nutrition absorption (Woods et al., 1988; Smith and Smith, 1990).

The second hypothesis is that it developed from a *de novo* formation when the haustorium was still in the process of developing (Kim et al., 2014). Arabidopsis resistance protein RPMW8.2 was found to specifically target the EHM where it promotes haustorial encasement formation and onsite accumulation of H_2_O_2_ and triggers hypersensitive response (Wang et al., 2009; Ma et al., 2014; Berkey et al., 2017). It was also established that RPMW.2 precise targeting to EHM involves an EHM-oriented specific trafficking pathway (Wang et al., 2013; Kim et al., 2014). These findings indicated that the EHM is a critical host-pathogen battlefield and not only a cross-border trafficking. It was also demonstrated that the EHM is fixed at the haustorial neck and physically detachable from the papilla and plasma membrane (Berkey et al., 2017). The EHM and plasma membrane were suggested to be probably two unique membranes of different origins (Berkey et al., 2017), because RPW8.2 was exclusively present in the EHM (Wang et al., 2009) and there were no eight plasma membrane-localized proteins in the EHM (Koh et al., 2005). Recent research has also shown that the endoplasmic reticulum membrane and the endoplasmic reticulum heterogeneous membrane have similar properties. However, the EHM does not depend on normal secretion, which suggests that an unconventional secretory channel within the endoplasmic reticulum may supply the essential materials (Kwaaitaal et al., 2017). Isolated *E. pisi* haustorial complexes (haustoria encased in the EHM) have been studied by the development of monoclonal antibodies. There was one antibody that was able to identify the 62 kDa protein in the haustorial plasma membrane starting at the neckband area (Mackie et al., 1991). *U. fabae* plasma membrane vesicles from spores and germlings were found to have severalfold H*^+^*-ATPase activity than vesicles from haustoria (Struck et al., 1996). This suggests that the haustorial H^+^-ATPase plays a crucial function in nutrient absorption, presumably *via* activating carrier proteins for solute transport. One putative permease (PIG2) was only found in *U. fabae* haustoria plasma membranes (Hahn et al., 1997a). It was therefore hypothesized that in rust haustoria, H^+^-ATPase and transport proteins such as amino acid permeases collaborate in the energy-driven absorption of plant compounds (Hahn et al., 1997a). It was also established that 1,3-glucans and chitin are localized on the haustorium surface, and chitin may be converted into chitosan by fungi so that they can avoid being identified by their hosts (Micali et al., 2011). Chitosan has less eliciting power than chitin does (Polonio et al., 2021). The mechanism by which the haustorium prevents chitin identification, however, remains to be a mystery. Recently, a systematic subcellular localization analysis of 14 members of *Arabidopsis thaliana* monosaccharide sugar transporter protein (STP) family suggested that an endoplasmic reticulum-localized sugar transporter AtSTP8 maybe recruited to the EHM during haustorium biogenesis where it may assist sugar translocation across the haustorial interface by powdery mildew haustoria from Arabidopsis host cells (Liu et al., 2021). Transmembrane domain prediction showed that AtSTP8 possesses 12 transmembrane domains which conforms with a typical monosaccharide transporter structure (Doidy et al., 2012; Paulsen et al., 2019). It was also suggested that AtSTP8 is an energy-dependent wide monosaccharide transporter with a preference for glucose (Liu et al., 2021).

## Molecular physiology of the haustorium

### Gene expression

High-throughput sequencing has permitted extensive studies of gene expression in non-model species, increasing knowledge of haustorial cell gene expression. In order to discover the molecular physiology of the haustoria, efforts have been made to isolate the haustoria in rust and powdery mildew (Gil and Gay, 1977; Hahn and Mendgen, 1992; Cantrill and Deverall, 1993; Catanzariti et al., 2006). Due to the complexity of haustorial isolation techniques, only a few transcriptome studies of rust and powdery mildew haustoria have been reported so far. Transcriptome studies established that, rust haustoria are extremely active in acquiring amino acids, carbohydrates, phosphate, and nitrogen (Hahn et al., 1997a; Voegele, 2006). The vast majority of genes in rusts that are haustorially expressed play essential roles in the synthesis of proteins, generation of energy, and metabolic processes. Furthermore, several transcripts were found to encode nutrient transporters or secreted proteins (Yin et al., 2009). These studies, on the other hand, only looked at a small portion of the genes that are expressed in the haustorium. The haustorium comprises a typical balance of organelles and nuclear genes. One of the most numerous and uniquely expressed genes in rust haustorium encodes a hexose transporter (HTX1; Voegele et al., 2001). This gene was expressed in leaf haustoria but not in *in vitro* infection structures. *U. fabae* haustoria strongly expressed two genes involved in vitamin B1 production (Sohn et al., 2000). This is a co-factor for enzymes involved in carbohydrate, amino acid, and biosynthesis processes. Work on this rust fungus revealed that the haustorium is extremely metabolically active and vital in nutrient absorption (Voegele et al., 2001). Differential screening of cDNA libraries from purified *U. fabae* haustoria provided further evidence for plasma membrane specialization. Hahn and Mendgen (1997) found at least 31 *in planta*-induced genes (*PIGs*), single or low-copy number genomic genes that code for proteins involved in amino acid transport, thiamine biosynthesis, metallothioneins, cytochrome P-450 mono-oxygenases, and short-chain dehydrogenases. *PIG* genes are highly expressed in infected leaf haustoria but not in germlings (germinated spores) or infection structures created on artificial membranes. Studies on cDNA microarrays showed enhanced expression of glycolysis (energy) and protein production genes in *B. graminis* infected barley (Both et al., 2005, 2005a). In the latter fungus, a partial proteome of haustoria showed significant representation of proteins implicated in protein metabolic pathways and energy production (Godfrey et al., 2009), and a comparative proteomic analysis confirmed higher protein and energy metabolism in haustoria (Bindschedler et al., 2009). Four thousand and four hundred and 58 transcripts were annotated from *Pst* haustoria using a Percoll gradient (Garnica et al., 2013). Most of the identified genes were associated with pathogen proliferation and metabolism, including biosynthesis of thymine, sugar transporters, and cell wall modification enzymes. Digital expression analysis showed 295 predicted secreted proteins overexpressed in haustoria (Garnica et al., 2013).

Another transcriptome analysis found that, in haustoria, 3,524 genes were upregulated relative to urediospores and germ tubes (Garnica et al., 2013). Functional categories uncovered by gene annotation and pathway analysis help them understand how differentially expressed genes operate in haustoria formation and host-pathogen interactions. Biotrophic colonization requires haustoria, as shown by the presence of ATP and TCA-related genes being upregulated in *Pst* (Garnica et al., 2013). Jakupović and colleagues found higher expression of *in planta*-induced genes involved in metabolic processes in haustoria than in other structures or stages of *U. fabae* (Jakupović et al., 2006). *Pst*ˍ*22758*, a ubiquitous heat shock protein containing a DnaJ domain, may be involved in protein folding, unfolding, transport, and degradation in haustoria (Qiu et al., 2006; Düppre et al., 2011). Cell wall biogenesis and DNA replication were shown to be increased in germinated urediniospores in a transcriptome study of RNAs extracted from *Pgt* haustoria and urediniospores (Upadhyaya et al., 2014). In another transcriptomics study, extracellular cell wall modifying enzymes like chitinases had a higher abundance in the haustorial transcriptome of *U. appendiculatus* (Polonio et al., 2019; Sharma et al., 2019). It was shown that enzymes that change extracellular cell walls may be detected in the haustorial transcriptomes of *E. pisi, G. orontii, and P. xanthii*. Overexpression of two chitin-binding/hydrolase/glycosylase proteins was found in the haustoria *of E. pisi*, while expression of a putative chitin lytic polysaccharide monooxygenase was found in *P. xanthii* haustoria (Polonio et al., 2019; Sharma et al., 2019). This suggests that these enzymes are important for haustorial physiology.

### Haustorium nutrient uptake and metabolism

**Figure 2:**
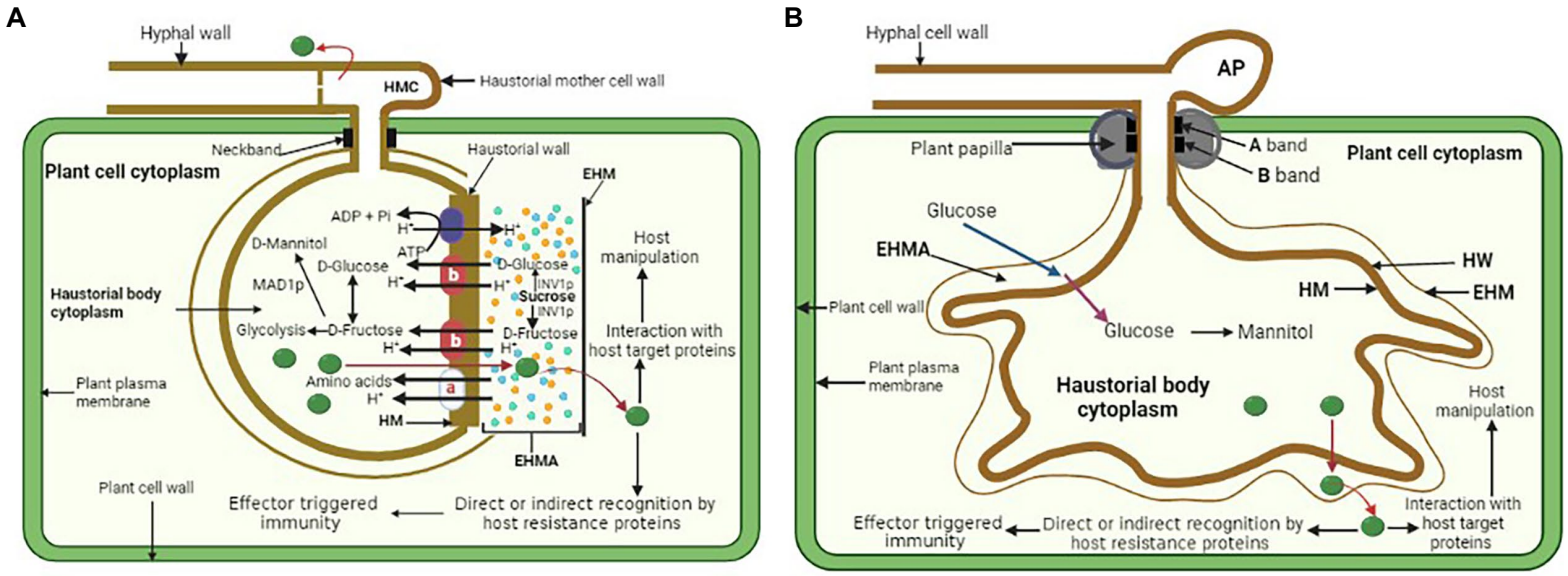
**(A)** General illustration of a rust fungus (dikaryon) haustorium and a proton symport model for the active uptake of amino acids and hexoses and redistribution from an infected leaf cell into a rust haustorium. EHMA, extrahaustorial matrix; EHM, extrahaustorial membrane; HM, haustorial plasma membrane. Haustorial plasma membrane H1-ATPase drives the uptake of nutrients by supplying protons. **a**—amino acid transporters AAT1 and AAT2; **b**—hexose transporter HXT1. Symport carriers utilize the resulting proton gradient between EHMA and haustorial cytoplasm to actively uptake amino acids and sugars. **(B)** General illustration of powdery mildew haustorium and its functions. HW, haustorial cell wall. Glucose passes through the EHM by facilitated diffusion (blue arrow) into EHMA where it is then actively transported (purple arrow) through the HM into the haustorial body cytoplasm. The glucose in the haustorium is converted to mannitol and then moves out to facilitate further mycelium growth. Both rust fungi and powdery mildew haustorium also secrete effectors (green) into the apoplast, including the EHMA and may pass through the EHM before entering the cytoplasm of the host plant where they are directly or indirectly recognized by the host disease resistance proteins resulting in effector triggered immunity, or they may target host proteins to manipulate host metabolism.

Fungal haustoria are feeding structures that transfer sugars and amino acids into the fungal cells. A few fundamental questions about the haustorium’s function remain unanswered. These include identifying pathways for bidirectional transport (nutrient import and effector export), pathogen and host proteins at the haustorium–host cell contact, and regulatory genes that determine haustorial identity. Detailed assessment of putative nutrient absorption processes is hindered by the fact that fully developed haustoria are generated only *in planta* and their purification leads to loss of function (Hahn and Mendgen, 1992). Hence, haustoria have been mostly studied cytologically. Cytological and molecular investigations unraveled indirect evidence of haustoria’s function in nutrient absorption (Gil and Gay, 1977). Sugar transporters and potential amino acid transporters in the structure suggest a function in nutrition absorption. An active transport mechanism is likely required to maintain high rates of carbon fluxes toward pathogens from the host leaves, since haustoria serve as potent sinks for metabolites. The transfer of nutrients (glucose, fructose, amino acids) from the EHMA into the haustorium is powered by the generation of a proton gradient across the haustorial plasma membrane by a membrane H1-ATPase (Szabo and Bushnell, 2001). Establishing a proton gradient is achievable because the EHMA is a sealed compartment surrounded by the EHM on the plant side, the HM on the fungal side, and the neckband prevents it from communicating with the apoplast (Szabo and Bushnell, 2001; Voegele et al., 2001). The haustorial H^+^-ATPase plays a crucial function in nutrient absorption, presumably *via* activating carrier proteins for solute transport. Electrochemical gradients may be generated by this enzyme, which combines ATP hydrolysis with proton extrusion (Figure 2A). Rust fungi lack crucial processes (nitrate and sulfate assimilation), which explains their reliance on nutrient intake from their hosts (Duplessis et al., 2011; Kemen et al., 2015). *E. pisi* powdery mildew has an ATPase activity that is linked to the haustorial plasma membrane, but not to the EHM, implying that the host cell has no control over solute fluxes (Baka et al., 1995). While the EHM enclosing monokaryotic haustoria displayed ATPase activity, the non-invaginated region of the host plasma membrane did not. As a result, the monokaryon’s filamentous haustorium seems to be less remarkable than the dikaryon’s haustorium (Baka et al., 1995).

Due to their ability to be separated from infected plant tissue, rust fungus haustoria provided the opportunity for the first study of haustorial gene expression in *U. fabae* (Hahn and Mendgen, 1997). The findings of this study uncovered genes that produce a hexose transporter known as HXT1 (Voegele et al., 2001) as well as three amino acid transporters known as AAT1, AAT2, and AAT3 (Mendgen et al., 2000; Struck et al., 2002, 2004). Immunolocalization studies indicated that HXT1 and AAT2 are at the haustorial plasma membrane (Mendgen et al., 2000; Voegele et al., 2001). The amino acid transporters AAT1 and AAT3 have selectivity for the amino acids L-histidine and L-lysine, as well as L-cysteine, L-methionine, and L-leucine (Struck et al., 2004). On the other hand, biochemical experiments demonstrated that AAT1 and AAT3 function as proton-dependent transporters with preferences for histidine/lysine and leucine/methionine/cysteine, respectively (Struck et al., 2002, 2004). These experiments provided the first evidence that haustoria are involved in the process of nutrient absorption. Homologs of haustorial AAT genes were found in different rust fungus (Hacquard et al., 2011; Garnica et al., 2013). Plasma membrane H^+^-ATPases are an essential component in the process of active nutrient absorption in both fungi and plants (Sondergaard et al., 2004). It would seem that the HXT1 sugar transporter is a symport carrier that derives its energy from H^+^-ATPase. Mendgen and colleagues provided a molecular analysis of the gene and its regulation, a functional characterization of HXT1, and exquisite cytological evidence that demonstrates HXT1 is localized solely in the haustorial plasma membrane (Voegele et al., 2001). It was proven by Voegele and colleagues that the HXT1 sugar transporter has a selectivity for D-glucose and D-fructose. This confirms that glucose and fructose, and not sucrose, are the major sugars that are imported by haustoria (Voegele et al., 2001). In addition, the authors demonstrated that the transfer of glucose takes place *via* a process known as proton symport. These findings provide credence to the hypothesis that nutrient transport at the haustorial interface is governed by a proton symport model (Figure 2A). Monokaryotic and dikaryotic haustoria, as shown by the location of AAT2p, showed identical molecular features while having significant morphological variations (Mendgen et al., 2000). Only the distal section of the monokaryotic haustorium and the dikaryotic haustorial body distal to the haustorial neck were found to have the putative amino acid transporter (Hahn et al., 1997a). The boundary of the fungal membrane that is delineated by the neckband is not the location where molecular differentiation takes place; rather, it takes place across domains of the membrane that are not morphologically distinct. Expression of AAT2p appears to occur only late after penetration of the host cell, particularly in monokaryotic haustoria, with negligible further lateral diffusion of the transport protein after that (Mendgen et al., 2000). However, in addition to its primary function of absorbing and using amino acids, the haustorium also plays a significant part in the metabolic processing of sugar. *In planta*-induced genes that are also induced in the biotrophic mycelium were identified from a cDNA library that was unique to the haustorium of the broad bean rust fungus, *U. fabae*. Thiamine, a cofactor for numerous metabolic enzymes, may increase resistance gene expression, and is involved in plants’ H_2_O_2_ pathway (Boubakri et al., 2012). It was observed that all haustorium-forming pathogens acquire their thiamine from the host since they lack a biosynthetic pathway. This indicates that thiamine plays a critical role in both the metabolism of the haustorium and the formation of the hyphae (Kemen et al., 2011). Two genes involved in thiamine biosynthesis, known as *THI1* and *THI2*, were discovered to have the highest levels of expression in haustoria (Sohn et al., 2000). Together, they made up less than 5% of the mRNA detected in haustoria (Staples, 2001). The *THI1*-encoded protein was shown to have a strong preference for localization in the haustorial cytoplasm, as determined by immunocytology. Thiamine diphosphate, often known as vitamin B1, is a cofactor that is required by a number of enzymes involved in the metabolism of carbon and amino acids. Metallothioneins eliminate oxygen radicals by scavenging them (Ruttkay-Nedecky et al., 2013). Therefore, silencing *Pst*ˍ*16188* may inhibit thiamine uptake from the host and thiamine metabolism in haustoria, which is consistent with obligate biotrophic fungus (Xu et al., 2020). Based on these findings, the haustoria are an important part of the main metabolic process (Sohn et al., 2000; Xu et al., 2020).

*PIG2* is homologous to fungal amino acid permeases that act as symport carriers, transporting substrates using the energy of a proton gradient provided by a plasma membrane H^+^-ATPase. There was an upregulation of the hydrolytic activity of a plasma membrane ATPase PMA1, in haustorial microsomal vesicles compared to germ tubes and ungerminated urediniospores (Struck et al., 1996). PMA1 molecular characterization established its autoregulation point and function in the process of nutrient absorption from host cells (Struck et al., 1998). PMA1 and MFL maltose transporter were both activated late in the fungal growth process (Weßling et al., 2012), but the absence of hexose transporters in the haustoria of *B. graminis* showed that powdery mildews make use of very few sugar transporters (Godfrey et al., 2009; Polonio et al., 2021). This may imply that, in contrast to rust haustoria, powdery mildew haustoria may make use of a variety of sources of carbohydrates. A major facilitator superfamily (MFS) sugar transporter was found to be among the top 50 expressed genes in a recent transcriptome study of *P. xanthii* haustoria. This study suggested that the powdery mildew fungi can use different methods for carbohydrate uptake (Figure 2B; Polonio et al., 2019). In addition to hexose and amino acid transporters, other nutrient transporters that are found in the haustoria have been characterized. *Pst* haustorial sulfate transporter (Yin et al., 2009), or the inorganic phosphate transporter among the top 50 expressed genes in the haustoria of Arabidopsis powdery mildew (Weßling et al., 2012), also showed that sulfur and phosphorus are taken up by the haustoria. The authors went on to demonstrate that this sugar transporter is localized in the haustorial membrane rather than in the membranes of the intercellular hyphae. This provided the first direct proof that sugar absorption takes place in the haustorium and indicated that it may be the sole site. Fourteen metabolism-related genes that were highly expressed in haustoria were silenced by host-induced gene silencing (Xu et al., 2020). It was shown that seven genes are involved in infection since silencing them in plants caused changes in the growth and development of *Pst*. The seven genes may have a role in the differentiation of cells, the development of pathogens, as well as the production of carbohydrates, amino acids, and thiamine (Nowara et al., 2010).

Oligopeptide transporters (OPTs) are plasma membrane-localized transport systems involved in the import of oligopeptides (Lubkowitz, 2011). Different transcriptomic studies found that rust OPT genes are highly expressed *in planta* (Duplessis et al., 2011; Garnica et al., 2013; Lorrain et al., 2019). The *Pst* genome comprises of 12 amino acid and 7 sugar transporter genes, including two hexose transporters identical to the *HXT1* gene in *U. fabae* (Zheng et al., 2013). In addition, *Pgt* has 21 OPT genes, compared to 5 to 16 in other basidiomycete fungi (Duplessis et al., 2011), indicating greater peptide absorption in rust fungi. The enlarged transporter families in rust fungus and upregulated expression indicate functions in nutrient accumulation. *Pst* transcriptome sequencing found a gene encoding a transporter with strong homology to an S-methylmethionine permease (Garnica et al., 2013). This transporter was exclusively expressed in haustoria compared to germinated spores and may be important for sulfur absorption. However, genes encoding amino acid biosynthesis and metabolism enzymes were also found, showing possibility of metabolism of ammonia and amino acids from host plants. Recently, a comparative genomic analysis of a transportome (a complete collection of transporters in a given genome) was performed in Pucciniales and compared with other fungi in the Dikarya (Guerillot et al., 2022). It was found that the transportome of fungi in the order Pucciniales is distinguished by gene family expansions associated with metal transport and most notably, oligopeptide transport. Intriguingly, OPT genes are not constitutively expressed throughout the whole life cycle of poplar rust fungus *M. larici-populina*; instead, they are expressed either in the spores infecting each alternative host or directly during biotrophic growth and proliferation inside the hosts. The high expression of many OPT in *Pst* isolated haustoria, together with amino acid transporters clearly confirmed that the specialized structure serves as a nutritional hub during pathogen-host interactions (Garnica et al., 2013; Lorrain et al., 2019). OPT genes exhibited dynamic expression patterns throughout the rust fungus life cycle, and especially during infection of the poplar host tree, suggesting a specialization for nitrogen and sulfur acquisition through oligopeptides transportation from the host during biotrophic growth (Guerillot et al., 2022). *In vitro* analysis of the bean rust *U. fabae* infection structures identified various extracellular proteases including metalloproteases (Rauscher et al., 1995). The proteases were suggested to release oligopeptides from degrading proteins within this compartment. The source of oligopeptides transported by rust fungi during their interactions with the hosts however remains a mystery. Therefore, it would be very interesting to try to determine the flux of the oligopeptides derived from the host plant and the material that serves as a source for such oligopeptides. Although OPTs are mainly responsible for the transport of oligopeptides, it cannot be ruled out that some specific OPTs may convey perception of signals during the interactions (Lubkowitz, 2006).

Other rusts, such as *Uromyces appendiculatus* and *Phakopsora pachyrhizi* (Link et al., 2014) and *Pgt* (Duplessis et al., 2011; Upadhyaya et al., 2014), have comparable haustorial transcriptomes, indicating they use similar processes to utilize host-derived resources. In contrast, rust fungal genomes lack nitrate/nitrite transporters and nitrate reductases in the NH_4_^+^ assimilation pathway (Duplessis et al., 2011; Zheng et al., 2013). This indicates nitrate absorption inability and amino acid absorption from host cells. Since the host provides the activities that are needed for mandatory biotrophy, the lack of sulfite reductase in *Pgt* shows that there is no sulfate assimilation pathway in mandatory biotrophy (Duplessis et al., 2011). Recently, the invertase PsINV was studied in wheat rust *Pst* and exhibited great sucrose hydrolysis efficiency and boosted wheat infection expression (Chang et al., 2017). Other genes from *U. fabae* that have to do with metabolism, like glucokinase GLK1p, mannitol dehydrogenase 1 (MAD1p), and NADH^+^ dependent D-arabitol dehydrogenase (ARD1p), have shown how this rust fungus stores and uses carbon (Voegele et al., 2005; Voegele and Mendgen, 2011). Homologs of these genes were discovered in other rust fungi and exhibited strong expression after infection, validating the processes of glucose absorption in rust fungus (Hacquard et al., 2010; Duplessis et al., 2011, 2011a; Garnica et al., 2013; Zheng et al., 2013). No sucrose transporter has been found in rust fungus, suggesting that hexose transport may be necessary in haustoria (Duplessis et al., 2011). Furthermore, findings from powdery mildew feeding experiments (Sutton et al., 1999) and rust fungi studies (Voegele et al., 2001; Voegele, 2006; Duplessis et al., 2011; Voegele and Mendgen, 2011; Zheng et al., 2013; Chang et al., 2017, 2020), collectively suggest that these pathogens mainly uptake hexose as the major carbohydrate source in the EHMA, where the hexose is either directly transported from host cells and/or converted from sucrose to hexose by a fungal invertase secreted into the EHMA (Liu et al., 2021). However, analysis of key haustorial genes of rust fungi only cannot enable conclusions regarding haustorial metabolism.

Biotrophic fungal pathogens are thought to strategically manipulate sugar transport in host cells to facilitate their access to carbohydrates. It was previously demonstrated that rust fungi or powdery mildew infection might cause an increased capacity for sugar acquisition in the infected host cells (Sutton et al., 2007; Chang et al., 2013). The increased sugar capacity may be due to enhanced activity of existing transporters or synthesis of new transporters in the infected host cells, which ultimately results in an increase in the amount of glucose available to the invading biotrophic fungal pathogens (Doidy et al., 2012). A sugar transporter *TaSTP6* was upregulated during rust infection and contributed to the susceptibility of wheat, most probably by enhancing cytoplasmic hexose concentration (Huai et al., 2019). Furthermore, a hexose transporter *Ps*HXT1 was recently found to have typical features of an MFS symporter with 12 membrane-spanning segments (Chang et al., 2020). *Ps*HXT1 was shown to be different from other rust fungal glucose transporters that have been characterized so far, and it only shared 26% similarity with *Uf*HXT1. *Ps*HXT1 was confirmed to be localized on the plasma membrane and this showed that it could function as a transporter like *Uf*HXT1 (Voegele et al., 2001). In addition, *Ps*HXT1 biochemical characterization using *Saccharomyces cerevisiae* mutant strain lacking 20 hexose transporters revealed that it has a substrate preference of glucose. This was also further confirmed by competition experiments which showed that *Ps*HXT1 only has a high affinity for glucose (Chang et al., 2020). Silencing of *PsHXT1* significantly restricted normal growth and development of *Pst* during wheat infection, resulting in reduced disease symptoms and fungal biomass. These findings clearly demonstrated that *Ps*HXT1 is a glucose-proton symporter (Chang et al., 2020). Taken together with the previous *Ps*INV study (Chang et al., 2017), it can be ascertained that sugar starvation does not only impair *Pst* invasive growth and development, but also slackens pathogen proliferation and virulence without possible muddle with signaling effects. Genes/proteins involved in sugar uptake are promising targets for disease control novel strategies because they are very conserved compared to effectors (Oliva and Quibod, 2017; Chang et al., 2020).

### Haustorial effectors

The fact that rusts and powdery mildews contain a high amount of transcripts encoding putative secreted proteins (Weßling et al., 2012; Garnica et al., 2013; Link et al., 2014; Polonio et al., 2019; Sharma et al., 2019; Xu et al., 2020), suggests that the haustorium might directly release effectors into the host cells, highlighting its importance not only in nutrients acquisition but also in pathogenesis, such as in the manipulation of host physiology and defense responses (Duplessis et al., 2012; Garnica et al., 2014; Polonio et al., 2021). For rust and powdery mildews, haustoria produce, secrete, and distribute virulence components, called effectors, that affect host physiology and immunity (Jones and Dangl, 2006). Several transcriptome studies focused on haustorial proteins to find haustorial effectors, and most of the identified proteins were novel candidates (Garnica et al., 2014; Link et al., 2014; Tao et al., 2017; Polonio et al., 2019; Elmore et al., 2020; Xu et al., 2020). Transcriptomic and genomic data from rust fungi found RTP1 homologs in at least 13 species, suggesting that this protein may play a role in biotrophic interactions (Pretsch et al., 2013). In the genomes of *Pgt*, *Pst*, *M. larici-populina*, and *M. lini* (Cantu et al., 2011; Duplessis et al., 2011; Nemri et al., 2014), approximately 8% of the predicted proteomes match the potential effectors that meet these requirements. Infection tissue-specific transcriptomes of these pathogens (Duplessis et al., 2011; Cantu et al., 2013; Garnica et al., 2013) and other rusts, notably *U. fabae* (Link and Voegele, 2008), have found multiple *in plant*-expressed effectors. Recently, haustoria-specific transcriptome data found 58% of the effector complement expected *in planta* in *Pgt* (Upadhyaya et al., 2014), supporting the hypothesis that the haustorium is the principal source of effector proteins. Proteome studies established that *Pt* has six effector protein signatures (peptidase and proteases, glucan-1,3-glucosidase and chitinases, protein disulfide isomerases, subtilisin-like serine proteases, cyclophilins, and carboxypeptidase; Song et al., 2011). *Pst* haustorial transcriptome analysis found an excess of cysteine-rich proteins among potential haustorial effectors and the majority of the secreted proteins produced in haustoria were expressed in a distinct manner from how they were expressed in germinating spores (Garnica et al., 2013). This is consistent with the fact that the majority of effector candidate genes are expressed in the haustoria, which is where they have the ability to operate directly by influencing the activities of host cells. A monoclonal antibody in mice was created using purified haustoria isolated from *Pt*-infected leaves. Purified haustoria contained 1,192 proteins, including 140 candidate secreted effector proteins (Rampitsch et al., 2015). In the haustorial transcriptome of *Pgt*, 520 secreted proteins were found, including 430 haustorially elevated secreted proteins and 90 genes expressed in germinated spores and haustoria as well as in haustoria alone (Cuomo et al., 2017). Research including transcriptome sequencing of isolated haustoria of stripe rust CYR31 race discovered 1,197 secreted proteins in haustoria, 69 of which suppressed cell death in tobacco and 49 decreased callose deposition in wheat, showing their critical roles in *Pst* infection (Xu et al., 2020). Six hundred and thirty-five candidate effectors of *P. triticina* were discovered through the RNA-seq of the interaction between *P. triticina* and wheat (Zhang et al., 2020).

Recent transcriptomics studies on powdery mildew haustoria established that there were some exclusively haustorially secreted proteins that included a SnodProt1 protein and many other putative adhesion proteins. The proteins had many ribonucleases and were also thought to be involved in protection against reactive oxygen species, phosphorous acquisition, and haustorial accommodation (Polonio et al., 2019; Sharma et al., 2019). Knockdown of some of the genes enhanced infection by powdery mildew (Sharma et al., 2019). Based on these findings, there may be a connection between the role of haustorium, its secreted proteins, and the proliferation of the pathogen. Since Y/F/WxC proteins account for 20% of the *B. graminis* haustorial transcriptome, it may be deduced that, this domain may play the key role in the physiological processes of powdery mildew (Polonio et al., 2021). The majority of the fungal haustorium cell’s proteins are either host-translocated or function at the EHMA since the fungal haustorium cell is located inside the plant cell. The presence of transcripts encoding putatively secreted proteins in rust and powdery mildew haustoria suggests that the haustorium is involved in the release of effectors directly into host cells, underlining the importance of this structure not only for nutrient uptake but also for how effectors enter plant cells, which is unknown (Polonio et al., 2021). RTP1 and Avr proteins indicate a class of rust effectors that are transported into host cells *via* haustoria, some of which could become targets of immune receptors. The first fungal proteins to enter host cells were RTP1ps from *Uromyces* spp (Kemen et al., 2005). The proteins were localized in host cells and then in the nucleus, suggesting a specific transport mechanism to their final location. Four different *M. lini* Avr proteins were successfully transported into their host cells (Rafiqi et al., 2010). All of these *M. lini* Avr proteins encode tiny secreted proteins that are produced in haustoria and found in the cytoplasm of the host, showing that they are delivered during infection. This was confirmed by direct visualization of the AvrM effectors (Rafiqi et al., 2010). Because AvrM and AvrL567 were expressed by tobacco cells, which were targeted to the plant secretory system, this work provided evidence that certain effectors may be taken up into the host cytosol regardless of a specific pathogen delivery mechanism. This was demonstrated by the fact that both of these effectors accumulated in the cytosol. AvrM is a membrane-binding protein that consists of two subunits, each of which has a hydrophobic surface patch that is essential for pathogen-independent internalization (Rafiqi et al., 2010; Ve et al., 2013). However, the mechanisms by which biotrophic fungal plant pathogens deliver their effectors into host cells are still a mystery.

## Conclusion and perspectives

The growth of biotrophic fungal pathogens needs an important structure, the haustorium, formed inside the cells of a living host plant. The haustorium is essential for nutrient acquisition, as well as secretion and delivery of effectors into the host cells. The development of transformation techniques, methods for isolating haustoria and intracellular hyphae, genome sequencing, and EST analysis help us to understand the function of haustorium in biotrophic fungal pathogens. Many genes involved in the production of energy, host defense suppression, and pathogenicity are expressed in haustoria, and gene expression analysis data has indicated that this structure is the root of biotrophic fungal pathogens. Nevertheless, there is limited knowledge on the signaling pathways involved in the development of haustoria and intracellular hyphae; the involvement of the fungus and the plant in assembly; the molecular foundation for their activities; and how they establish and sustain a biotrophic interaction with a suitable host. In the near future, the rapidly expanding collections of genes obtained through large-scale sequencing projects, as well as the application of gene-expression profiling and functional genomics, will make it possible to conduct in-depth research on a variety of regulatory and functional phenomena. Future studies need to focus on the mechanisms underlying EHM biogenesis and functions, whether immune signaling occurs at the pathogen interface or not, and also EMH manipulation by the pathogens for their own benefit. There is still a great deal of fundamental issues unanswered. How does pathogen infection affect EHM content? How are symbiotic interfaces formed, and what signals drive their formation? How exactly does the movement of metabolites and minerals through the membranes of donor cells take place? Which characteristics (for example, the secretion of enzymes and toxins, interface components, and signaling mechanisms) differentiate biotrophic pathogens from endophytes, hemibiotrophs, and necrotrophs? In order to guarantee a steady supply of nutrients from the host plant, how can fungal symbionts cause metabolic sinks to form at the sites of infection? In the process of sink induction and the symbiotic differentiation of the fungus, what functions do sugars and enzymes that metabolize sugar (like invertases) play? Answers to these questions will reveal complicated molecular and cellular processes at the haustorial interface. Our understanding is limited to transporters strongly expressed in various phases of rust fungi, leading us to believe that they are essential nutrient transporters. Future research should also identify all nitrogen transporters. Also, rust fungi’s nutrition signaling and amino acid absorption regulatory components must be identified. Thus, functional investigations of predicted haustorial proteins are essential. These components might help us to understand how biotrophic fungal plant pathogens evolved to affect their hosts.

## Author contributions

JM and WY: conceptualization and literature search. JM: writing—original draft preparation. JM, WY, LZ, and NZ: writing—review and editing. WY: supervision and funding acquisition. All authors contributed to the article and approved the submitted version.

## Funding

This work was funded by the Natural Science Foundation of China (nos. 301871915 and 32172367), National Key R&D Research Program of China (nos. 2013CB127702 and 2017YFD0201707), Natural Science Foundation of Hebei Province (no. C2020204071), and Modern Agricultural Industry System of Wheat Industry in Hebei Province (no. HBCT2018010204).

## Conflict of interest

The authors declare that the research was conducted in the absence of any commercial or financial relationships that could be construed as a potential conflict of interest.

## Publisher’s note

All claims expressed in this article are solely those of the authors and do not necessarily represent those of their affiliated organizations, or those of the publisher, the editors and the reviewers. Any product that may be evaluated in this article, or claim that may be made by its manufacturer, is not guaranteed or endorsed by the publisher.
